# Prevention of inadvertent perioperative hypothermia as a quality indicator in anesthesia: an eight-year experience from 2014 to 2022

**DOI:** 10.1186/s12871-026-04022-4

**Published:** 2026-06-22

**Authors:** Alper Tunga Dogan, Feyzi Artukoglu, Omur Ercelen

**Affiliations:** Department of Anesthesiology and Reanimation, American Hospital (Amerikan Hastanesi), Nisantasi, Istanbul, Turkey

**Keywords:** Perioperative hypothermia, Quality indicator, Anesthesia safety, Quality improvement, PDCA cycle, Ppatient safety, Temperature management

## Abstract

**Background:**

Inadvertent perioperative hypothermia remains one of the most common yet undertreated complications in anesthesia practice. Although evidence-based guidelines for its prevention have long been available, a significant gap persists between existing knowledge and clinical implementation. This study reports an eight-year institutional experience in which perioperative hypothermia was adopted as a quality indicator and managed through a collaborative framework between the anesthesiology department and the hospital quality management unit.

**Methods:**

A single-center, retrospective quality improvement study was conducted between May 2014 and September 2022. Adult patients who underwent surgery lasting more than four hours under general anesthesia were included. Postoperative body temperature was measured by tympanic infrared thermometry upon admission to the recovery unit; values below 36 °C were recorded as hypothermia. A Plan, Do, Check, Act (PDCA) cycle was employed in four phases: problem identification and baseline data collection (2014), awareness and education interventions (2014–2015), structural and technical improvements including provision of tympanic thermometers to all operating rooms and standardization of forced-air warming protocols (2015–2016), and continuous monitoring with periodic reinforcement (2016–2022). Data were collected monthly during 2014–2016 and through a sampled audit of one representative month per quarter from 2017 onward. Trends were analyzed using a Cochran, Armitage test and grouped binomial logistic regression, and process stability with a statistical process control chart.

**Results:**

A total of 1,902 patients were evaluated over the study period, of whom 348 (18.3%) were found to be hypothermic postoperatively. The annual hypothermia rate was 32.5% in 2014, falling to 25.9% in 2015 and 15.2% in 2016 following the educational and structural interventions. From 2017 to 2022, rates stabilized between 9.7% and 15.1%, representing an approximate 3.2-fold reduction from baseline. The decreasing trend was statistically significant (Cochran, Armitage *p* < 0.001; odds ratio 0.806 per year, 95% CI 0.762, 0.852). Intermittent spikes were observed in isolated quarters (e.g., 32.4% in the third quarter of 2019), but targeted re-education sessions led to prompt correction. The lowest annual rate recorded was 9.7% in 2021.

**Conclusion:**

Systematic identification and monitoring of perioperative hypothermia as a quality indicator, coupled with relatively straightforward interventions (education, equipment provision, and ongoing surveillance), was associated with a substantial and sustained reduction in hypothermia rates over eight years. Active collaboration between the quality management unit and the anesthesiology department, with a designated physician serving as quality representative, appeared central to sustaining these gains. These findings suggest that a structured, PDCA-based quality improvement approach may help bridge the gap between established guidelines and day-to-day clinical practice, even without large-scale resource investment; as a single-center experience, this should be regarded as a working hypothesis requiring multi-site confirmation.

**Supplementary Information:**

The online version contains supplementary material available at https://doi.org/10.1186/s12871-026-04022-4.

## Introductıon

Approximately 230 million patients worldwide undergo major surgical procedures requiring anesthesia each day. Surgery and anesthesia are inherently complex processes that carry the potential for serious complications and mortality. The World Health Organization (WHO) and the World Federation of Societies of Anaesthesiologists (WFSA) have emphasized that access to safe anesthesia is a fundamental right and have published international standards applicable across all resource settings [1, 2]. These standards call for the establishment of a patient safety culture, continuous process monitoring, and the adoption of evidence-based practice.

Inadvertent perioperative hypothermia is defined as a drop in core body temperature below 36 °C occurring at any point from the preoperative period through postoperative recovery [3]. General anesthesia suppresses thermoregulatory mechanisms, and the operating room environment further predisposes patients to heat loss. Prevention of inadvertent perioperative hypothermia is addressed by major clinical guidelines, including the United Kingdom National Institute for Health and Care Excellence guideline CG65 [4], the American Society of PeriAnesthesia Nurses (ASPAN) evidence-based normothermia guideline [5], the TARD guideline [3], and standards for basic anesthetic monitoring [6], reflecting broad consensus that active warming and perioperative temperature monitoring reduce hypothermia-related complications. The consequences of unintended hypothermia are well documented and include prolonged duration of action of hypnotic agents and neuromuscular blocking drugs, increased transfusion requirements, cardiac complications, delayed recovery from anesthesia, elevated surgical site infection risk, postoperative nausea and vomiting, and higher hospital costs [3, 7, 8].

Quality management and continuous improvement have become cornerstones of modern anesthesia practice. Close collaboration between the hospital quality management unit and the anesthesiology department, particularly when an anesthesiologist serves as a designated quality representative, plays a decisive role in the early detection and resolution of patient safety issues. Incremental, targeted changes in awareness and practice can, over time, produce lasting institutional improvements. The present study describes how inadvertent perioperative hypothermia was selected as a quality indicator at our institution, the interventions that were implemented, and the outcomes observed over the period from 2014 to 2022.

## Materıals and methods

### Study design and setting

This was a single-center, retrospective, observational quality improvement study conducted jointly by the Department of Anesthesiology and Reanimation and the Quality Management Unit at Amerikan Hospital between May 2014 and September 2022.

### Ethical approval

This study was approved by the Koç University Institutional Review Board (approval number: 2023.039.IRB1.010). As the study was based on retrospective analysis of routinely collected quality improvement data with no direct patient intervention, the requirement for individual informed consent was waived by the ethics committee.

### Patient selection and inclusion criteria

Adult patients who underwent surgical procedures lasting more than four hours under general anesthesia (a group at elevated risk of hypothermia) were included. All consecutive eligible cases in each audited period were included; there was no additional selection. Body temperature was measured by tympanic infrared thermometry at the time of admission to the postanesthesia care unit (PACU). A postoperative tympanic temperature below 36 °C was classified as inadvertent perioperative hypothermia [3].

### Data collection

Data were recorded prospectively by the quality management unit using standardized collection forms. For each reporting period, the number of patients with a postoperative body temperature below 36 °C, the total number of patients evaluated, and the hypothermia rate were documented. During 2014–2016, data were collected on a monthly basis; from 2017 onward, as the process stabilized and to balance the auditing workload, surveillance continued through a sampled audit of one representative month per quarter (typically February, May, August, and November). Reported quarterly figures from 2017 therefore correspond to these sampled audit months rather than to every eligible case in the quarter. The same inclusion criteria and outcome definition were applied throughout.

### Quality improvement process

The quality improvement process was structured around the Plan, Do, Check, Act (PDCA) cycle and proceeded through the following phases [9, 10].

#### Phase 1, problem identification (2014)

During routine meetings between the quality management unit and the anesthesiology department aimed at identifying areas for improvement in the surgical process, perioperative hypothermia was identified observationally as a probable, clinically important problem and selected as a priority quality indicator. A preliminary data-collection period and recurring quality meetings established the problem and planned corrective actions. In patients whose surgery exceeded four hours, tympanic body temperature was measured upon arrival at the PACU, and values below 36 °C were systematically recorded.

#### Phase 2, awareness and education (2014–2015)

In-house educational sessions were organized for the anesthesia team, operating room nurses, and support staff. These sessions covered the definition, risk factors, and complications of inadvertent perioperative hypothermia as well as preventive strategies. Educational content was based on the Turkish Society of Anesthesiology and Reanimation (TARD) Guideline [3] and the WHO-WFSA International Standards for a Safe Practice of Anesthesia [1, 2].

#### Phase 3, structural and technical improvements (2015–2016)

Before the programme, three tympanic infrared thermometers were shared across the twelve operating rooms and moved between rooms as needed and forced-air warming devices were present in all operating rooms but were not used for every patient. During this phase, a tympanic infrared thermometer was provided for every operating room to enable periodic intraoperative temperature checks and forced-air warming devices were additionally placed in the preoperative and postanesthesia care areas. Written protocols governing the routine application of warming were established (see Active Warming Protocol below). Operating room ambient temperature was maintained above 21 °C in accordance with the TARD guideline [3].

#### Phase 4, continuous monitoring and sustainability (2016–2022)

Hypothermia rates continued to be monitored on a regular basis. When periodic increases were detected, targeted refresher sessions and awareness meetings were convened. Regular meetings with the quality management unit were maintained, and the hypothermia prevention protocol was incorporated into the orientation program for newly appointed staff.

### Active warming protocol and equipment

A standardized institutional protocol (document KHC.F.6235.029; provided as Supplementary Appendix 1) was applied across the perioperative pathway, with the target of maintaining core temperature between 36 °C and 37 °C. Preoperatively, patients were informed that the environment is cooler than at home and reminded that they could request additional blankets; although active prewarming was not performed, a passive insulating cover was placed over each patient on reception in the preparation area. Temperature was recorded by the tympanic or oral route, and except in emergencies patients were not transferred to the operating room until the temperature was at least 36 °C. Intraoperatively, operating-room ambient temperature was kept above 21 °C before the patient entered the room, the operating table was warmed before arrival, and active warming was delivered with a forced-air warming system (3 M Bair Hugger), initiated at 38 °C and increased to 43 °C according to the tympanic temperature trend and discontinued above 37 °C. Blanket coverage (upper-body or lower-body) was selected case by case according to the surgical field. Intravenous fluids were warmed to 37 °C and irrigation fluids to 38–40 °C. The protocol required intraoperative temperature monitoring at approximately 30-minute intervals; however, only the PACU-admission temperature was retained as the study outcome. Postoperatively, temperature was recorded on PACU admission, and patients were not transferred to the ward until the temperature exceeded 36 °C.

### Feedback mechanism

Feedback operated through the hospital-wide quality structure. the Quality Management Unit shared quality metrics monthly with departmental quality representatives, who relayed the relevant findings to their departments. For the perioperative pathway, the operating-room nursing and anesthesia-technician leaders maintained direct contact with the unit, and the anesthesiology quality representative coordinated corrective measures. When a deviation was detected, a root-cause analysis was performed and the whole team was briefed, with corrective reminders issued. Feedback was therefore provided at the level of the operating room, surgical specialty, and professional group, rather than to individual clinicians monitoring their own performance over time. Baseline staff knowledge of the protocol was not formally assessed, and adherence to specific process measures (for example, the proportion of patients receiving forced-air warming or warmed fluids) was not tracked. Only the outcome, PACU-admission temperature, was monitored.

### Temperature measurement

Body temperature was measured using tympanic infrared thermometry. A single, standardized infrared device (Braun ThermoScan) was used throughout, and the devices were calibrated at regular intervals by the hospital biomedical engineering department. In line with the TARD guideline, the tympanic route was preferred for postoperative measurements [3]. Measurements were taken at the time of PACU admission and repeated at 15-minute intervals when indicated.

### Outcome measures

The primary outcome measure was the proportion of patients with a postoperative body temperature below 36 °C (perioperative hypothermia incidence) in each reporting period. This was calculated as: (number of patients with postoperative temperature < 36 °C / total number of patients evaluated) × 100.

### Statistical analysis

Period-specific incidence was expressed as a proportion with 95% confidence intervals calculated by the Wilson method. The temporal trend in annual proportions was assessed with the Cochran, Armitage test for trend, and a grouped binomial logistic regression was fitted with calendar year as a continuous ordinal predictor, reported as the odds ratio per year with 95% confidence interval. A segmented (interrupted time-series) logistic regression of the monthly and quarterly series distinguished the active-intervention era (2014–2016) from the sustainability era (2017–2022). Process stability was evaluated with a Shewhart p-control chart using 3-sigma control limits computed separately for the two eras to account for the variable number of patients per period. As a secondary analysis, patient-level data assembled from the audited months (1,759 patients) were analysed with a multivariable binomial logistic regression of postoperative hypothermia on calendar year, surgical duration, emergency status, and surgical specialty. PACU-admission temperature was additionally analysed as a continuous variable. A two-sided *p* < 0.05 was considered significant. Analyses were performed in Python 3 (statsmodels and scipy). An artificial intelligence language model (Claude, Anthropic) was used to assist with statistical analysis and English language editing of the manuscript.

## Results

Over the study period (May 2014 to September 2022), a total of 1,902 patients whose surgery exceeded four hours under general anesthesia were evaluated. Of these, 348 (18.3%) had a postoperative body temperature below 36 °C.

### Annual hypothermia incidence

**Fig. 1:**
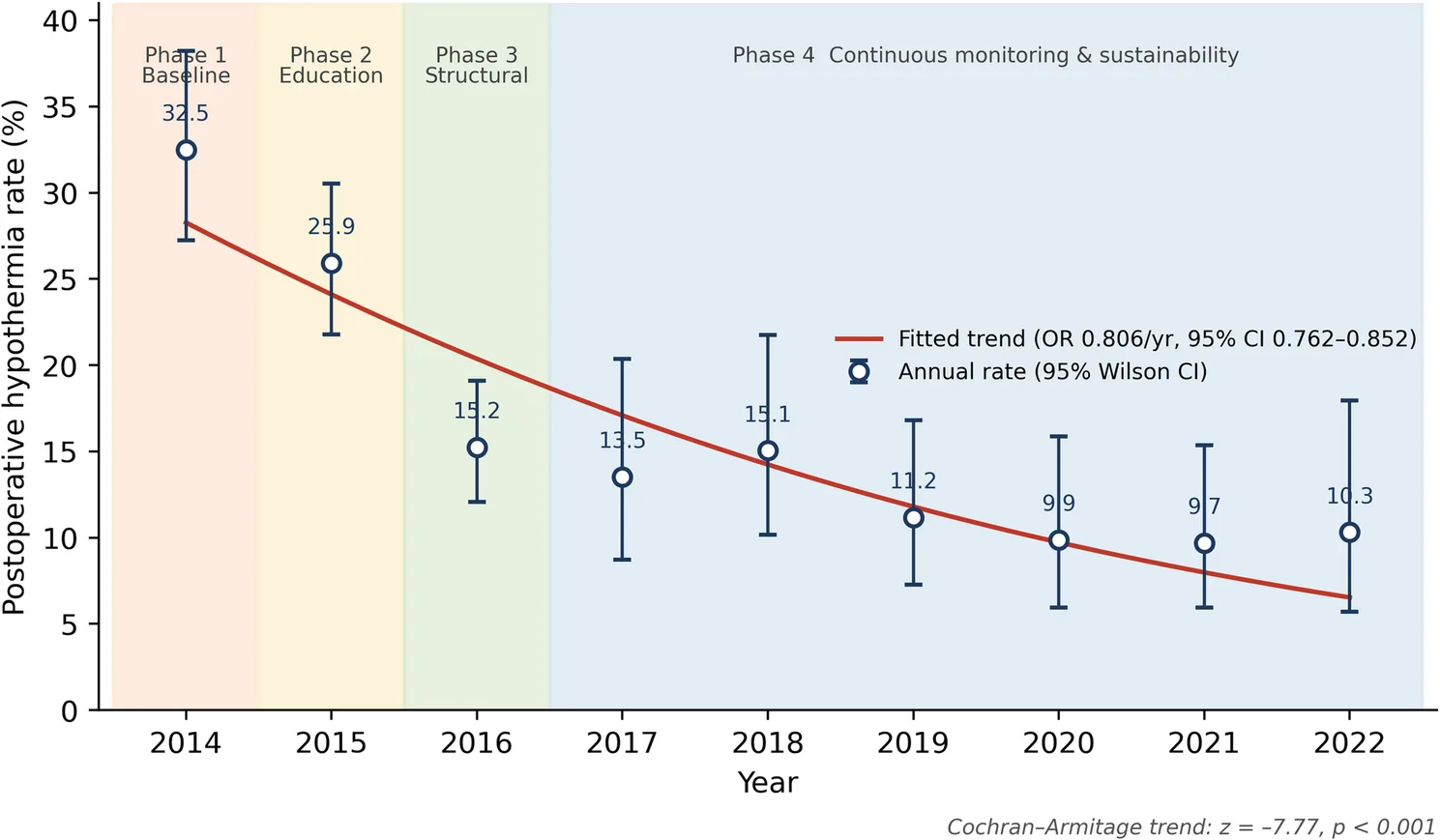
Annual postoperative hypothermia incidence (2014–2022) with 95% Wilson confidence intervals and the fitted logistic trend (odds ratio 0.806 per year, 95% CI 0.762, 0.852). Shaded bands indicate the phases of the quality-improvement programme. Cochran, Armitage trend test z = − 7.77, *p* < 0.001

In 2014, the year in which quality improvement efforts were initiated, 90 of 277 monitored patients (32.5%) were found to be hypothermic postoperatively. Following the introduction of educational and awareness interventions, the rate fell to 25.9% in 2015. After structural improvements were implemented, the rate declined further to 15.2% in 2016. In subsequent years, ongoing monitoring and periodic refresher sessions maintained the rate within the 10 to 15% range. The lowest annual rate, 9.7%, was recorded in 2021 (Table 1). The decreasing trend across calendar years was highly significant (Cochran, Armitage test, z = − 7.77, *p* < 0.001). In the grouped binomial logistic regression, each successive year was associated with a 19% reduction in the odds of hypothermia (odds ratio 0.806 per year, 95% CI 0.762, 0.852, *p* < 0.001); the odds in 2014 (32.5%) were approximately 4.5-fold those in 2021 (9.7%) (odds ratio 4.49, *p* < 0.001). The annual incidence and the fitted trend are shown in Fig. 1.

**Table 1:** Annual postoperative hypothermia incidence with 95% confidence intervals (2014–2022)

Year	Hypothermic (*n*)	Evaluated (*n*)	Rate (%)	95% CI (Wilson)
2014	90	277	32.5	27.2–38.2
2015	99	382	25.9	21.8–30.5
2016	61	400	15.2	12.1–19.1
2017	18	133	13.5	8.7–20.4
2018	22	146	15.1	10.2–21.8
2019	19	170	11.2	7.3–16.8
2020	14	142	9.9	6.0–15.9
2021	15	155	9.7	6.0–15.4
2022*	10	97	10.3	5.7–17.9
Total	348	1902	18.3	—

*2022 data cover the first three quarters only. *CI* Confidence interval (Wilson method)

### Statistical process control and time-series analysis

**Fig. 2:**
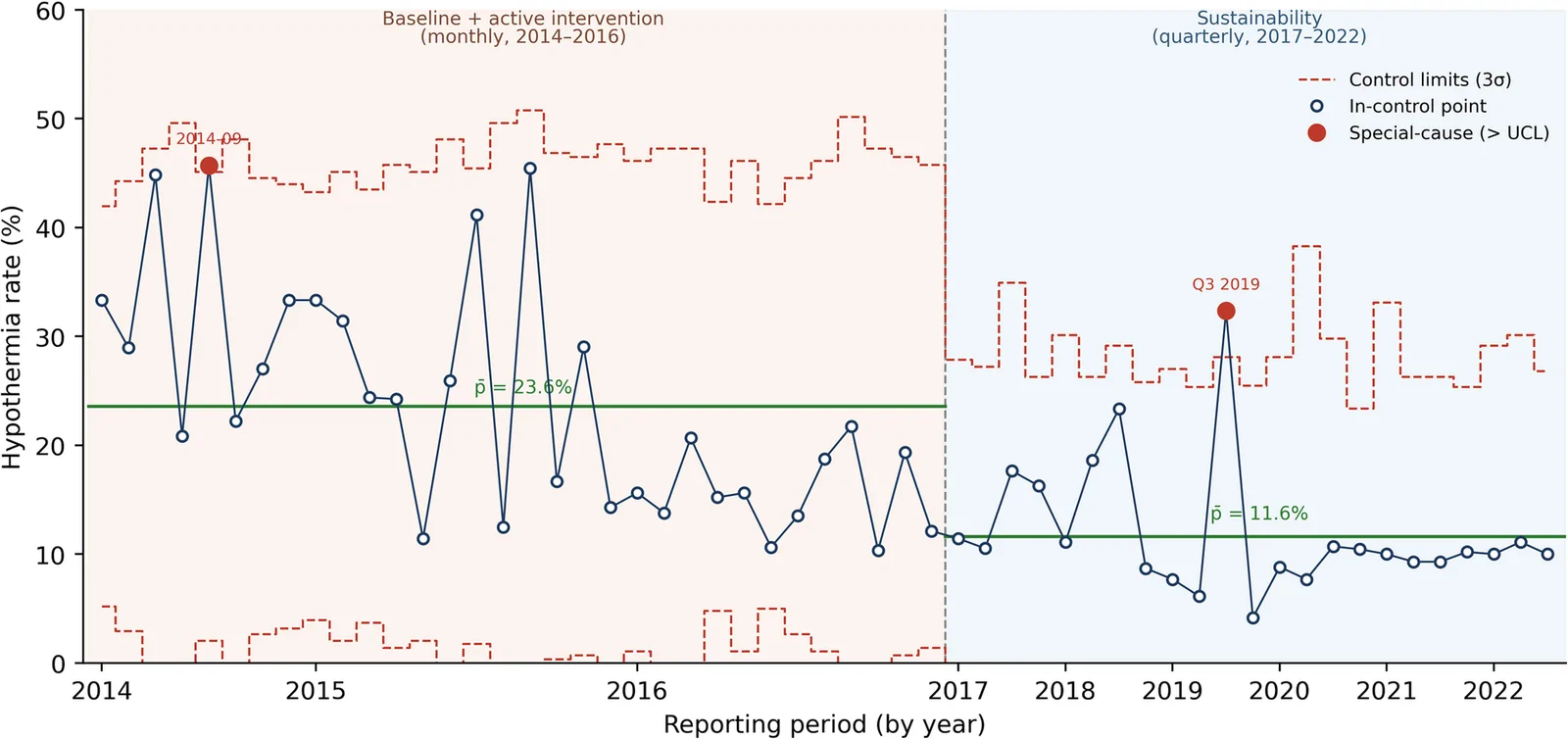
Shewhart p-control chart of the hypothermia rate by reporting period (monthly 2014–2016; quarterly 2017–2022), with period-specific 3-sigma control limits computed separately for the baseline/active-intervention and sustainability eras. The centre line (mean proportion) fell from 23.6% to 11.6%. Red markers denote special-cause points exceeding the upper control limit

On the p-control chart (Fig. 2), the mean hypothermia proportion fell from 23.6% during the baseline and active-intervention era (2014–2016) to 11.6% during the sustainability era (2017–2022), consistent with a sustained downward shift in the process rather than random fluctuation. Four monthly points exceeded the upper control limit during the early period (all in the summer months of 2014 and 2015), whereas only a single special-cause signal occurred during the entire sustainability era, the third quarter of 2019 (32.4%), which returned within control limits in the following quarter (4.2%) after a targeted refresher session. In the segmented interrupted time-series model, the downward trend during the active-intervention era was significant, whereas neither the level change nor the slope change at the 2017 transition was significant, indicating that the steep decline coincided with the active-intervention phases and that the lower rate was subsequently maintained rather than further reduced. The quarter-by-quarter pattern of the sustainability era, including the transient spikes and their correction, is shown in Supplementary Figure S1.

### Case-mix and sensitivity analysis

**Fig. 3:**
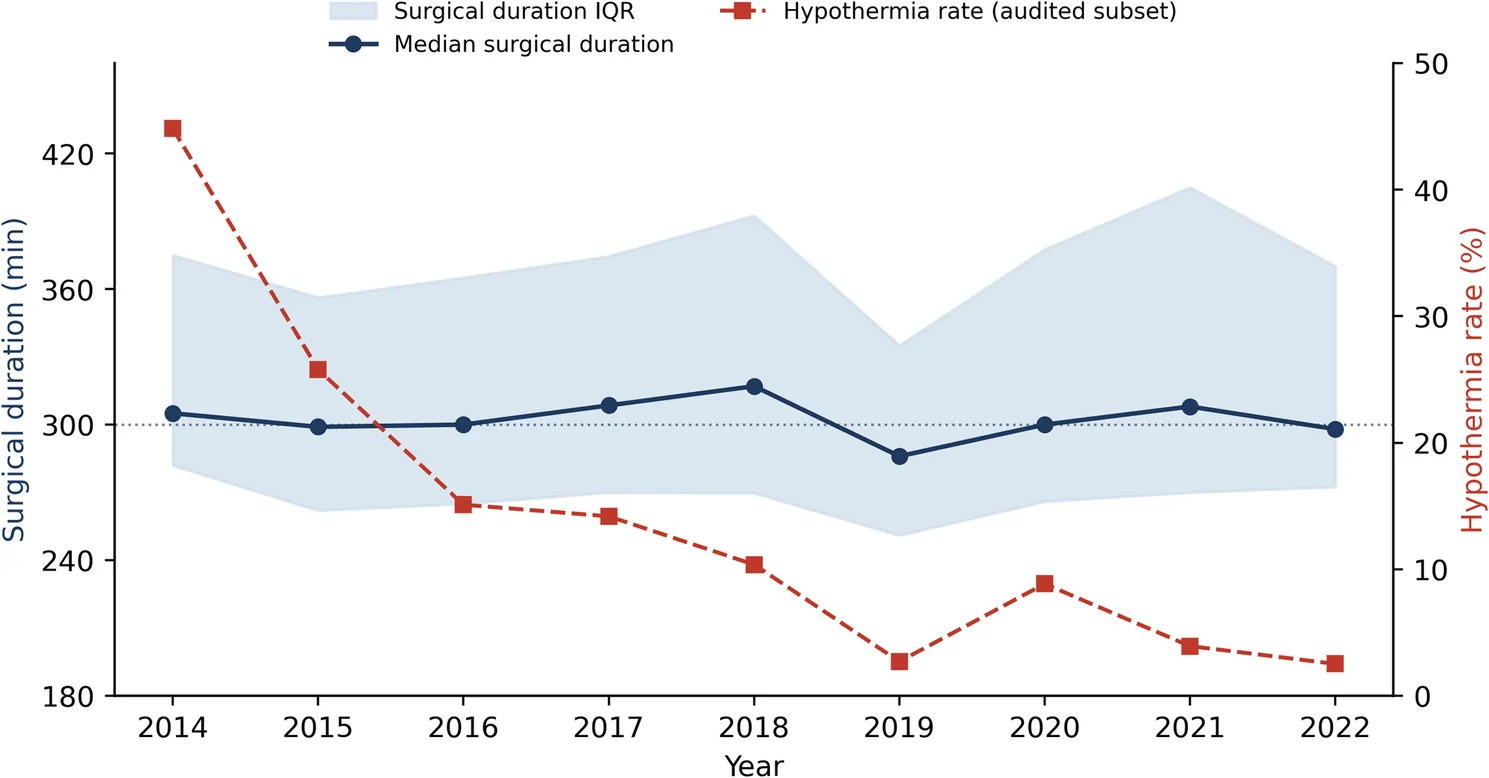
Median surgical duration with interquartile range (left axis) and postoperative hypothermia rate in the audited subset (right axis) by year, 2014–2022. Surgical duration remained stable at approximately 300 min while the hypothermia rate declined, indicating that the reduction is not attributable to a shift toward shorter procedures

For a subset of the audited periods, individual patient-level records could be retrieved from the quality database (1,759 patients). These were used for the descriptive and sensitivity analyses below and do not correspond exactly to the aggregate series in Table 1, owing to incomplete patient-level capture in some periods (Table 2). Median surgical duration remained stable at approximately 300 min throughout the period (range of annual medians 286 to 317 min), and the proportion of emergency procedures remained low (0 to 7%), indicating that the audited population did not shift toward shorter or lower-risk procedures over time (Fig. 3). In the multivariable logistic regression, the odds of hypothermia decreased with calendar year (unadjusted odds ratio 0.688 per year, 95% CI 0.635, 0.745); after adjustment for surgical duration, emergency status, and surgical specialty, the year effect was essentially unchanged (adjusted odds ratio 0.694 per year, 95% CI 0.640, 0.754, *p* < 0.001), and surgical duration was not an independent predictor within this cohort of long procedures. PACU-admission temperature, analysed as a continuous variable (available for 1,165 patients), increased over the period (+ 0.036 °C per year, *p* < 0.001; 36.36 °C in 2014–2016 versus 36.54 °C in 2020–2022).

**Table 2:** Characteristics of the audited cohort by year (patient-level audited months)

Year	Audited months	Patients (*n*)	Median duration, min (IQR)	Emergency, *n* (%)
2014	1	29	305 (282–375)	2 (6.9)
2015	12	384	299 (262–356)	10 (2.6)
2016	12	404	300 (265–365)	13 (3.2)
2017	4	134	308 (270–374)	3 (2.2)
2018	4	164	317 (269–392)	3 (1.8)
2019	4	185	286 (251–335)	4 (2.2)
2020	4	147	300 (266–377)	3 (2.0)
2021	4	153	308 (270–405)	5 (3.3)
2022	4	159	298 (272–370)	0 (0.0)

Hypothermia rate in the audited subset is shown for description only; the primary analysis uses the full institutional aggregate series (Table 1). 2014 reflects a single audited month

### Overall trend

From the baseline rate of 32.5% in 2014 to 10.3% in the final year of the study (2022), hypothermia rates were reduced approximately 3.2-fold. The steepest decline occurred between 2014 and 2016, coinciding with the active intervention phases. Rates subsequently reached a plateau of roughly 10% and remained stable. The prompt identification and correction of periodic spikes was consistent with the value of the ongoing surveillance strategy.

## Dıscussıon

The present findings show that adopting perioperative hypothermia as a quality indicator and managing it through a structured collaboration between the anesthesiology department and the hospital quality management unit can yield meaningful and durable results with relatively simple, low-cost interventions. Reducing the postoperative hypothermia rate from 32.5% to approximately 10%, and maintaining that level for eight years, is consistent with the practical impact of a quality management approach on patient safety, although causation cannot be confirmed in an uncontrolled design. In a comparable initiative, Cates et al. reported that a quality improvement project raised temperature monitoring compliance from 10% to 90% in a pediatric dental operating room [11], and Hanna et al. demonstrated that a checklist and education program reduced neonatal perioperative hypothermia from 44.4% to 23.4% [12]. Most published quality-improvement initiatives for perioperative hypothermia, however, report short observation windows of only weeks to months; the principal contribution of the present work is its eight-year observation period, which shows that a reduction achieved during an active-intervention phase can be sustained over the long term through continued surveillance and feedback. To our knowledge, few perioperative hypothermia programmes have reported sustainability over this length of time, which is the main reason the full eight-year period is presented rather than a shorter window.

Anesthesiology occupies an unusual position in medicine as a specialty that has historically led the way in patient safety. The founding of the Anesthesia Patient Safety Foundation (APSF) in 1985 represented one of the earliest independent institutional efforts in healthcare devoted solely to patient safety [13]. The anesthesiologist is situated at the center of the operating room ecosystem, following the patient from preoperative assessment through intraoperative management to postoperative recovery. Harfaoui et al. noted that while technical and pharmacological advances have made it possible to perform increasingly long and complex procedures, these advances have also introduced new safety challenges [14]. Mathis et al. argued that anesthesiologists should look beyond the boundaries of the operating room, building interdepartmental collaborations, and emphasized a kaizen-style philosophy of continuous, incremental improvement [15].

Quality and safety are inseparable concepts in perioperative care. In a systematic review, Haller et al. identified 108 clinical quality indicators for anesthesia [16]. Wacker et al. pointed out that most perioperative quality indicators rest on low-to-moderate levels of evidence [17]. The 2018 update of the WHO-WFSA International Standards lists body temperature monitoring among recommended standards [2]. In the United States, perioperative temperature management is a reportable quality measure (Quality ID #424) under the Medicare Merit-based Incentive Payment System [18].

Inadvertent perioperative hypothermia continues to rank among the most frequent yet least monitored perioperative complications. Its reported incidence varies from 10% to as high as 80%. Wang and Deng identified advanced age, low body mass index, duration of anesthesia or surgery, preoperative hypothermia, and large-volume transfusion as leading risk factors [19]. Complications include surgical site infection [20], coagulopathy and increased blood loss [7], morbid cardiac events [8], shivering, prolonged emergence, and increased hospital costs [3, 19].

One of the more notable findings of our study was that the act of systematically measuring and reporting hypothermia rates appeared to produce improvement even before structural interventions were introduced, consistent with the principle that what gets measured gets managed. Munday et al. argued that how clinicians think about the problem may matter more than the technical details of any particular warming protocol [21]. Along similar lines, Cargile et al. suggested that appropriate management of operating room ambient temperature is the single intervention with the greatest potential to reduce intraoperative hypothermia risk [22]. Tympanic infrared thermometry was used because it is practical and non-invasive in a high-throughput setting. However, infrared tympanic readings are an indirect estimate of core temperature, are not recommended for this purpose by some guidelines [4], tend to read higher than oesophageal or pulmonary-artery measurement, and may underestimate the true incidence of hypothermia. Because a single standardized device (Braun ThermoScan) was used and calibrated at regular intervals by biomedical engineering, and the same method was applied consistently throughout, measurement drift is unlikely to explain the temporal trend. Nonetheless, absolute incidence figures should be interpreted with this measurement limitation in mind.

Evidence-based guidelines and protocols for perioperative hypothermia prevention, including those issued by NICE [4], TARD [3], ASPAN [5], and AORN, have been available for many years, yet adherence in routine clinical practice remains suboptimal. Heidke et al. identified equipment costs, increased workload, and low awareness as the principal obstacles [23]. This persistent evidence-practice gap underscores the critical role of hospital quality management units. The spike observed during 2019 Q3 (32.4%), and its rapid correction to 4.2% in the following quarter, offers a concrete illustration of how continuous monitoring and rapid feedback can work in practice.

Effective interdepartmental collaboration is a determining factor in the success of quality improvement programs. In our experience, having an anesthesiologist serve as the designated quality representative was critical for the rapid detection of emerging problems and for ensuring that implemented measures remained in place. Hill et al. argued that anesthesiologists are ideal quality partners for hospital administrators [24]. Tung et al. underscored that quality improvement is a professional obligation for anesthesiologists [25].

### Limitations

Several limitations should be acknowledged. First, the single-center, retrospective, before-and-after design without a concurrent control group means that the contribution of the programme cannot be fully separated from secular trends or other concurrent changes, and causation cannot be established. Second, because the study covered an eight-year period, unmeasured changes in surgical case-mix (procedure type and complexity, duration beyond the four-hour threshold, open versus minimally invasive approach, elective versus emergency status), in anesthetic practice (opioid use and opioid-sparing techniques, volatile versus total intravenous anesthesia, depth-of-hypnosis titration and processed-EEG monitoring, neuromuscular blockade and ventilation strategy), in perioperative workflow and operating-room environment (patient preparation, prewarming, ambient temperature, transfer routines), and in fluid and hemodynamic management (fluid volume, warmed fluids, vasopressor use, anti-shivering medication) may all have influenced postoperative temperature independently of the programme, these were not systematically collected and remain unmeasured confounders, although adjustment for surgical duration, emergency status, and specialty in the patient-level cohort did not attenuate the temporal effect. Third, the outcome was measured only at PACU admission. Although the protocol required intraoperative monitoring, intraoperative values were not retained as study data, so the study reflects postoperative rather than intraoperative hypothermia. Fourth, postoperative temperature was assessed by tympanic infrared thermometry. Although a single standardized device (Braun ThermoScan) calibrated at regular intervals was used throughout, tympanic thermometry remains an indirect estimate of core temperature that reads higher than oesophageal or pulmonary-artery measurement and may underestimate the absolute incidence of hypothermia. Fifth, although the programme included education and refresher sessions, baseline guideline knowledge and adherence to process measures were not assessed, so the specific behavioural mechanisms mediating the improvement cannot be determined. Sixth, from 2017 surveillance used a sampled audit of one month per quarter, which reduced temporal resolution and means that sampled months may not fully represent every case in a quarter. Finally, clinical outcomes plausibly related to hypothermia (surgical-site infection, cardiac events, length of stay) were not assessed.

### Future directions

Future studies should incorporate clinical outcome measures, including surgical site infection, cardiac morbidity, and length of stay, alongside hypothermia incidence to permit cost-effectiveness analysis. Multicenter studies would allow comparison across institutional settings. Routine integration of preoperative warming (prewarming) deserves consideration; the meta-analysis by Uçak et al. confirmed its effectiveness [26]. Embedding automated temperature alerts into electronic health record systems could facilitate monitoring. All such initiatives would benefit from the collaborative framework between the quality management unit and the anesthesiology department described here.

## Conclusıon

This study demonstrates that systematic management of inadvertent perioperative hypothermia as a quality indicator, supported by education, equipment provision, and sustained monitoring, was associated with a meaningful and lasting reduction in hypothermia rates. The collaboration between the hospital quality management unit and the anesthesiology department, anchored by a physician quality representative and guided by a PDCA cycle, coincided with an approximately 3.2-fold decrease in hypothermia incidence over eight years, an association that persisted after adjustment for surgical duration, emergency status, and specialty. Periodic spikes were detected and corrected promptly, supporting the value of a continuous surveillance approach. These results suggest that quality improvement in anesthesia does not necessarily require large-scale investment. As a single-center experience, the transferability of this model should be regarded as a working hypothesis requiring multi-site confirmation; the adoption of similar models for other perioperative quality indicators and across different institutional contexts is worth evaluating.

## Supplementary Information


Supplementary Material 1.


## Data Availability

The datasets used and analyzed during the current study are available from the corresponding author on reasonable request.
