# Supplementary material for: Prevention of inadvertent perioperative hypothermia as a quality indicator in anesthesia: an eight-year experience from 2014 to 2022
Source: BMC Anesthesiol. 2026 Jun 22;26:544. doi: 10.1186/s12871-026-04022-4 (PMC13536770; doi:10.1186/s12871-026-04022-4)
Supplement: Supplementary file 1 [file 12871_2026_4022_MOESM1_ESM.docx]

**Supplementary Materials**

*Prevention of Inadvertent Perioperative Hypothermia as a Quality Indicator in Anesthesia: An Eight-Year Experience from 2014 to 2022*

# Supplementary Appendix 1. Institutional perioperative hypothermia prevention protocol

Institutional protocol for the prevention of inadvertent perioperative hypothermia (document KHC.F.6235.029), showing the preoperative, intraoperative, and postoperative algorithm and temperature thresholds. Adapted from the Turkish Anaesthesiology and Reanimation Society (TARD) Guideline for the Prevention of Inadvertent Perioperative Hypothermia, 2013.

# Supplementary Figure S1. Quarterly hypothermia rates during the sustainability phase

Quarterly hypothermia rates during the continuous-monitoring phase (2017–2022), based on the sampled audit month for each quarter. Red bars indicate quarters with rates exceeding 20%; orange bars indicate rates between 15% and 20%; blue bars indicate rates below 15% (normal range). The transient increases in 2018 Q3 (23.3%) and 2019 Q3 (32.4%) were each followed by prompt correction after targeted refresher sessions.
